# The role of serious games in the iManageCancer project

**DOI:** 10.3332/ecancer.2018.850

**Published:** 2018-07-11

**Authors:** Stefan Hoffmann, Stephen Wilson

**Affiliations:** 1 Promotion Software GmbH, Tübingen 72072, Germany; 2 University of Bedfordshire, Bedfordshire, Luton LU1 3JU, UK

**Keywords:** serious games, childhood cancer, iManageCancer, remission

## Abstract

Within the iManageCancer project, two serious games were developed, one for adults and one for children and adolescents. The adult’s game was developed by the University of Bedfordshire (UK), the kid’s game by Promotion Software GmbH (Germany). The aim was to support adult and young cancer patients with serious games to manage the impact of the disease on their psychological status, such as negative emotions, anxiety or depression, and motivate them to stay positive and to participate in social life [Patterson and Garwick (1994) *Ann Behav Med* **16** 131–142; Brennan *et al*. (2002) *J Consul Clin Psychol* **70** 1075–1085; Robinson *et al*. (2007) *J Pediatr Psychol* **32** 400–410]. Early demonstrators showed how important it is that players understand the meaning behind the game design. Improvements in the design of the games addressed that issue. Also, technical and use case issues were found that had a significant impact on the outcome. Both games interact with the iManageCancer platform, record game results and make them available for research. Two pilots are on the way and another evaluation cycle will follow.

## Introduction

The treatment and personal situation of cancer patients have dramatically changed over recent decades. The disease can now be managed in many cases as a chronic illness requiring long-term surveillance. The iManageCancer project reflects this development and provides a cancer disease self-management platform including a personal health record (PHR) (iphr.care) designed according to the specific needs of patient groups and focusing on the well-being of the cancer patient.

Also, two serious games became an essential element with an emphasis on psycho-emotional evaluation and encouragement to set self-motivated goals. One focuses on adults and was developed by the University of Bedfordshire (UK). The other game is for children and adolescents and was developed by Promotion Software GmbH (Germany), a company specialising in the development of serious games.

## The term ‘Serious Games’

Today, Serious Games are developed for many different purposes, and there are common misleading conclusions of the term. Mainly, the term ‘Serious Game’ has been around for many years in various forms and uses, ranging from military applications such as war games to any activity app that utilises the game-based design principles in conjunction with an objective that is not explicitly for fun or recreation. ‘Serious Games’ are not a specific game genre, and any type of game can be utilised in that context.

It has been suggested [1] that the term gained prominence after the American military released the video game America’s Army [2] in 2002 which was a first-person shooter intended to familiarise players with the US Army and serve as a recruitment tool.

In its current context, a Serious Game can be described as a game which has a primary purpose other than entertainment [3]. This does not mean that entertainment is unimportant for serious games. Entertainment still plays an important role to achieve these ‘serious’ purposes, because even in a serious game a game can be defined as an ‘activity engaged in for diversion or amusement’. Amusement makes the player go on, so only in this way can the purpose be fulfilled.

## Two games with different purposes

Both serious games for iManageCancer were developed separately with completely different gameplay aspects and purposes.

The target group for the serious game for adults is adults with a cancer disease. People in this target group have to reorganise their lives and may have to rethink their habits and their overall lifestyle. The idea behind the serious game for adults is to create a game that focuses on these issues, makes the players concentrate on these thoughts and encourages a learning transfer from the game into the player’s life, to encourage healthy habits and to promote critical, strategical thinking. To reach this goal, a game setting was created, which allows the player to develop his/her own avatar and his/her own city, where he/she rehearses living a well-balanced life between healthy habits and enjoyment.

The target group for the serious game for kids are children with cancer and also their relatives and friends. The impact of the disease on the psychological status of the target group is significant. The treatment process makes them passive participants in many ways, which may result in feelings of anger and helplessness. The idea behind the game is to make children and their supporters become active players in the treatment process, make them focus on a new target and push their mindset in the direction of attacking the real enemy, the cancer cells.

Another problem for children, especially while they are in the hospital, is their isolated situation. To improve this, social aspects were included, allowing family and friends to interact via game mechanics with the children.

The serious game for kids is implemented as a classical shooter game. The player is flying with a vessel from left to right, searching for cancer cells and eliminating them.

This article describes the rationale behind both games, how the game design and development process worked and how the games changed from the original idea to the game versions that were chosen for the two pilots within the iManageCancer project.

## Essential groundwork for the development process

Before developing the game prototypes, essential groundwork had to be done to make the basic decisions for the game design. Beyond the basic idea behind the games, the most important aspects to mention here are the use cases, the technical aspects and a review of references and several serious game effects described in the research literature.

## Technical aspects of development

Choosing a hardware platform for a game is always a decision that has to consider many aspects of the typical use case we foresee for a game. In our case, the hardware has to match the following criteria:

- The device can be used in hospitals
- The device can be made available easily for children
- The device is highly attractive for children and adults
- The device allows individual accounts and—permanent or temporary—access to the Internet.

That shows quite clearly that a PC or a PS4 console with a TV is not the proper device for our projects.

As a result of this analysis, both games were developed for mobile gaming platforms like tablets and smartphones, for the operating system Android. As a technical base, the Unity 3-D engine was chosen. This is a very powerful 64-bit game developing framework which allows the development of 2-D and 3-D games. The development works cross-platform. The software can be tested instantly on the PC and after this exported to the target platform Android. Unity 3-D allows a variety of target platforms and is planning to support future platforms, so there’s a high chance that the resulting games are future-proof.

The games were edited in C# and animated with the internal editor of Unity 3-D. For the serious game for kids, the editor was enhanced for improved editing processes of the game levels, enemy behaviour and more. 3-D models like the cancer cells were originally designed and iterated in Photoshop and then modelled, rigged (=prepared for animation) and animated in 3DS Max and ZBrush.

Both games were designed to establish a connection to the internet and exchange data with a game backend and the iManageCancer platform iphr.care. For the serious game for adults, there is also an offline version, which does not have the advantage of retrieving the patient’s data from the platform.

The serious game for kids allows communication between the devices, controlled by the iManageCancer platform. This is used for some fine social game elements described later in this article. This game was also developed to be played offline, with some kind of ‘going only when possible’ mode.

## Serious game effects

Research of the effect of serious games is still ongoing, facing the problem that it is nearly impossible to come to basic conclusions with the many facets which serious games can have. Even between similar games within one genre comparison is difficult (see references on ‘Re-Mission’), between different genres, it is nearly impossible [4]. It is assumed that the effectiveness of Serious Games is mixed, regarding the Design Elements chosen for the game [24], but actual meta-analysis confirms that serious games overall are more effective than conventional instruction methods [25].

Ke [5] pointed out that, consistent with the finding of previous gaming reviews, this analysis indicates that the empirical research on instructional gaming is fragmented by research variables (i.e. research purpose and methodology), administrative variables (i.e. learning setting), learner variables, procedural variables (i.e. game-based pedagogy) and game variables (e.g. game genre and media).

Some research results seem to be transferable, so meta-analysis points out that using narrative elements in a Serious Game has counterproductive effects [25]. As a result, we reduced narration to a minimum, but did not resign it completely.

For the serious game for kids, another effect was important. A common way to realise social learning in video games is to postulate the learning from others, e.g. with ‘models’ (characters and avatars) that are older than the player, to facilitate the adoption of correct behaviour [6]. The adaption method in the serious games for iManageCancer uses similar transfers, an idealised me and characters with an extremely positive attitude about their cancer treatment.

## Other relevant serious games

Several serious games are available which are relevant references for the design of the serious game for kids and the serious game for adults. Two games with a significant influence are described here.

### ‘Re-Mission’

**Figure 1.**
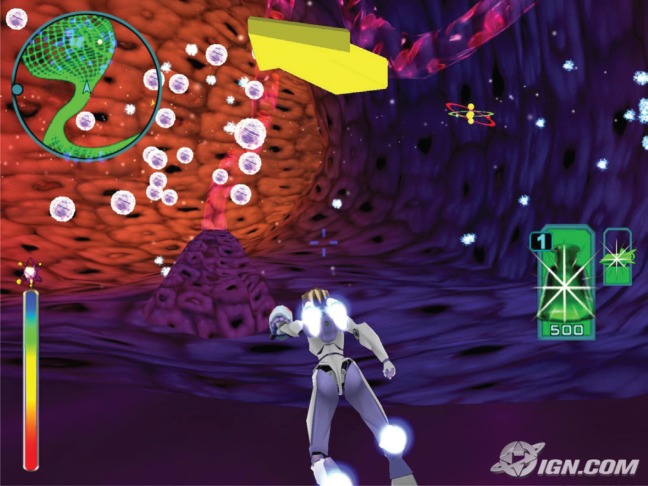
Screenshot of the game ‘Re-Mission’ shows an in-game situation.

‘Re-Mission’ (Figure 1) [7–9] is a serious game for children with cancer and for their relatives. The game explains what happens in a human body during different cancer diseases. The idea behind the game is: by killing the cancer cells with the hero, the user motivates himself or herself by fighting against the disease and recognising the importance of ingesting medicine. The game includes 20 stages with different kinds of cancer. It is a path-breaking game developed by many game developers, animators, cancer experts, biologists and psychologists with a 4.6-million-dollar budget.

The intervention with ‘Re-Mission’ significantly improved treatment adherence and indicators of cancer-related self-efficacy and knowledge in adolescents and young adults who were undergoing cancer therapy [23].

The serious game for kids in the iManageCancer platform motivates children to fight against cancer—like ‘Re-Mission’—and shows the way that medicine works, and how important daily medicine is. Moreover, the focus shifted from the educational part to a game with stronger motivational mechanisms. The graphics are more suitable for younger children, also the game has a coop mode and strong social components for better patient support.

### ‘Crusaders of the Lost Idols’

**Figure 2.**
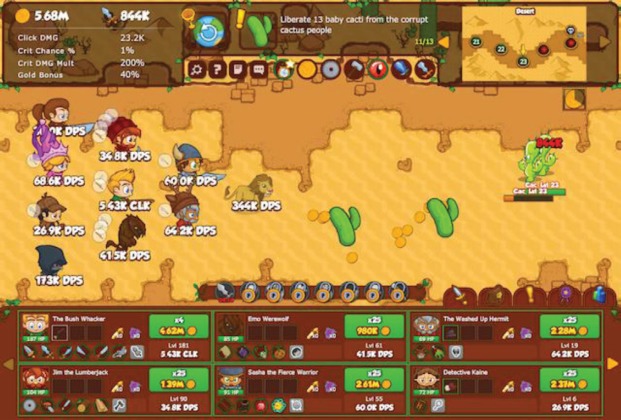
Screenshot of the game ‘Crusaders of the lost idols’ shows an in-game situation.

‘Crusaders of the Lost Idols’ (Figure 2) is a web-based game available from Kongregate [10]. This game is a side-scrolling click and drag adventure game. The game can also be left unattended, leaving the character to automatically execute its strategy. The mechanic of recruiting and strategically positioning allies can be combined with a design that does not involve real-time multiplayer tactics, providing an opportunity to populate the serious game for the adult player’s game with avatars and stats of friends and supporters to be used in a strategic manner.

## Objectives and game design basics of the serious game for adults with cancer

The serious game for adults aims to help users engage with the wider iManageCancer platform while placing them in a situation requiring critical thinking and strategy. The players are in the situation of reorganising their lives and may have to rethink their habits and their lifestyle.

The idea of the game is to encourage the player to proceed in this process and promote self-efficacy, i.e. the belief of the patients to be able to manage and to face their disease, also to support the patients in dealing with the psychological dimension of their disease, promote a healthier lifestyle and a better disease management. As pointed out in [11], two of six studies about self-management of the disease showed that serious game players benefit more than the control group [12].

To give the feeling to the player that he/she plays a relevant role in this game and also in the recuperation process, we put him/her in the role of a mayor, an authority figure (see [8]), who manages a small town where he/she helps residents with their cancer-related lifestyle problems. The user is invited to think critically and strategically in order to balance resources and time, while also viewing the issues surrounding his/her cancer from a different perspective. The game is intended to promote the concept that with a good management of a person’s cancer disease they can still be happy and achieve a sense of wellbeing. It also transmits the idea that all choices rely on continuously ongoing balancing processes.

The targets of the design of the serious game for adults were:

- (Concrete) Convey healthier lifestyles (balanced diet and appropriate physical exercise) and improve problem-solving skills (adjustment-oriented self-management approach)
- (Meta) To create a game that educates patients (and individuals) to engage in healthy behaviours while having fun
- (Meta) Improve self-efficacy in self-management skills through mastery and vicarious experience of the Avatar in the serious game.

The teaching method of the game is not to make medical recommendations or specifically advise the user about appropriate lifestyle changes, instead opting to provide examples of good lifestyle management within a controlled context using a fictional character. The game also provides an educational element through a mini-game which leverages a large set of health and lifestyle trivia questions in a quiz-style challenge. The mini-game is repeatable and is designed to dissuade the fictional cancer patient from unhelpful lifestyle choices.

## The serious game for adults step-by-step

From the first basic idea of a mayor of a town who provides a lot of public and personal decisions to the player, a first game design draft was formed. The following section describes the gameplay of the game step-by-step. The development process contained a lot of iterations and corrections of ideas that were not working. This learning is described in the section ‘Evolving design process in the game development phase’.

### Creating an avatar

**Figure 3.**
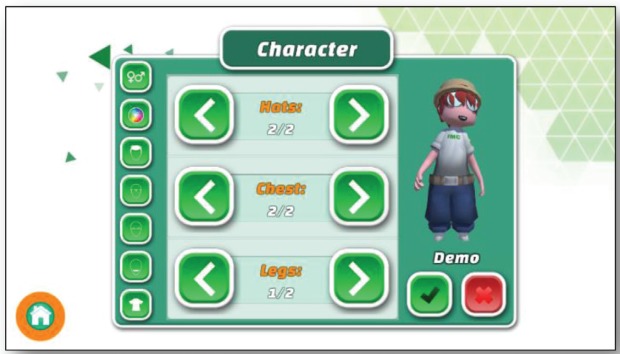
Screenshot shows the character customisation menu from the serious game for adults.

We wanted to give the player the possibility to find instantly an avatar for identification in the game. Therefore, the game starts with a process to create an individual avatar (Figure 3). This character finally represents the mayor character in the game world.

The game allows a lot of modifications of the avatar within the character generator including hairstyle, eyes, mouth, glasses, gender and clothing.

Once the character is individualised the player confirms and the mayor starts in the game world.

### Give the user progressive aims and missions

**Figure 4.**
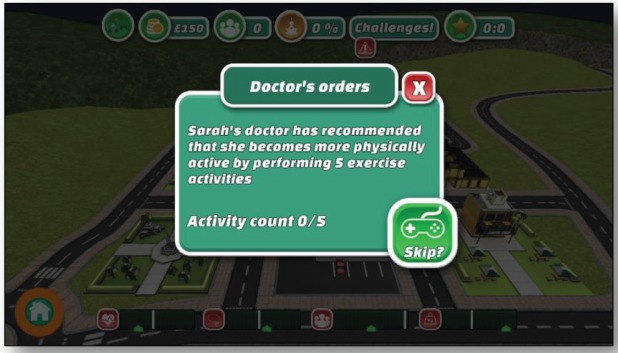
Screenshot shows a doctor’s order to the player in the serious game for adults.

Each game level gives the player an individual aim to achieve, presented by a character that players must assist (Figure 4). The user’s aim is to get this citizen’s wellbeing value to 100%.

Once the player proceeds in the game, new levels become unlocked and new challenges are given. This can be to complete a number of activities within the town or build a certain building.

### Put the user in a critical situation for a strategy of solutions

**Figure 5.**
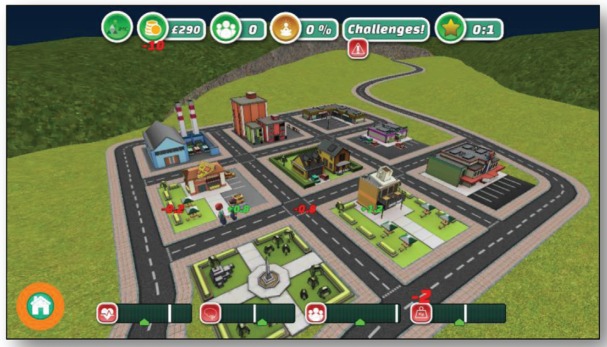
Screenshot shows a typical in-game situation in the serious game for adults.

Figure 5 shows the main view of the game which shows the city with all the buildings and streets. Also, the player character is wandering through the streets. This part of the game is shown in realtime 3-D graphics. The position of the camera can be changed at all times to show the buildings from a completely different perspective.

From here, the status can be seen, also the development of the city. Selecting a building or an estate offers the opportunity to develop the city or using the city’s infrastructure (like buying healthy food or junk food).

The player is faced with the challenge of balancing the citizen’s time and budget, they must consider the needs of their citizen and the current resources of the town to proceed on an optimal course to 100% wellbeing.

‘Poor’ choices can now lead to negative well-being, the goals and activities while not always being medically relevant to the user are designed to stimulate critical thinking about time and lifestyle.

## The integration of the serious game for adults into the ‘iManageCancer’ platform

The serious game for adults is not designed mainly as stand-alone software. Registered iManageCancer users can access a feature that utilises their physical activity data, this data is collected through other iManageCancer applications and allows the user to convert it in order to spend it within the game as a form of currency.

Currently, the feature allows the user to buy a random prize chance box, this box is exchanged for a spin on a prize wheel that yields a small beneficial prize in the game. The feature is intended to encourage a user to utilise more of the iManageCancer platform and potentially motivate the user to engage in more physical activity.

Registered users can also save their game data to a game backend service once they have logged in. This service allows for registered users to provide feedback and audit data which is forwarded through the backend service to their PHR within the iManageCancer platform. Further to basic game profile uploading and migration a user can also search for other people’s profiles and send them friend requests. This social mechanic is an asynchronous form of social play, a friend’s profile can be viewed in the game and there is a mechanic that allows the friend to occupy the user’s town providing a passive effect. The social profiles contain no medical data or information beyond their avatar’s current appearance and contact e-mail.

## Evolving design process in the game development phase

Early feedback was collected from workshops held by Tenovus Cancer Care, a Welsh cancer charity, and initiated by ecancer, a leading oncology education provider. This has led to several design changes not only of the interface but also of the core design mechanics.

By changing the role of the user to that of a supporter of a fictional cancer patient the confusion of many users was addressed regarding their role and the nature of the actions the game expects them to undertake.

These changes also addressed the issue of users trying to relate too closely to the challenges in the game—in many situations these challenges were contrary to their real-life situations resulting from cancer.

Other learnings from this feedback were that the target group and the use case became much more carved out. In fact, an information gap became visible between the knowledge of the player and the requirements of the game. To make the transfer of learning possible, hints and tutorials were integrated into the game.

There was also an insight that the individualisation of the avatar has a high significance for a certain type of player, especially for players who tend to the socialiser role in the scheme of the Bartle Test [13]. To support these players with socialiser emphasis, more customisation features like different clothes or hair colours were added to the game.

The next step in the development will be to prove if all the enhancements lead to a measurable improvement regarding the mentioned issues.

## Objectives and game design basics of the serious game for children and adolescents with cancer

One of the basic objectives of the serious game for kids is the improvement of self-efficacy, based on a transfer of learning in the game to the situation in reality. Transfer of learning is usually described as the process and the effective extent to which past experiences (also referred to as the transfer source) affect learning and performance in a new situation (the transfer target) [14]. So, the idea behind the design is to show children that fighting against cancer is an active process for the child. Children and relatives should be not only actively involved in fighting against cancer but also play a passive role in the treatment. The players travel in a virtual vessel through a human body and fight virtual cancer cells with different weapons that represent the therapeutic clinical tools against cancer. The intended transfer of learning is quite simple and understandable for younger children: that weapons exist and that patients can combat cancer if properly applied (which is specifically a figural transfer, as defined by Royer [20]).

Another objective of the design was to lower the child’s resistance against the treatment. There also might be aggressions against physicians and parents, because of the side effects of the treatment, and the game should help to lead these aggressions in the right direction, against the cancer cells. The method of childhood distraction is already recognised for reducing pain and anxiety with video games, with a better effect than with passive media consumption like watching cartoons. [15, 16].

The outcome of cancer treatments for children is very good, but the path to a cure is stony, with numerous physical side effects and psychological reactions. Much more than with adults, the psychological support includes the whole family. How a family responds to adversity influences the child’s responses and functioning, in a circular sequence of effects [17]. A positive relation has been found between parental distress and the distress in children, e.g. children of depressed mothers display a variety of internalising and externalising symptoms, above and beyond those displayed by children of non-depressed mothers [18]. Other links have been found between the anxiety of parents and the anxiety of children [19]. This leads directly to the insight that an enhancement of empowerment and resilience of a child with cancer would have a lack of effect without including family members.

As a result, the serious game for kids was designed with social features, allowing the family members to support the ill children and make them also an important part of the ‘in-game treatment’ in the game. This also addresses the isolation of the kids in the hospital, when they have limited contact with family and friends. The serious game allows supporters (family members and friends) to play and contribute to the game’s achievements for the children’s play success. The more the supporters play for the child, the more powerful the weapons against cancer cells become in the game of the child.

In addition to the already mentioned social features, the arena mode was designed to initiate social interaction between the patients and the game. This arena mode works as follows: play success of all players is measured week by week. If all the game results of all players summed up fulfil the goal for the current week, the arena opens and all players (if they took part or not) can fight a very nasty special cancer cell. Every player that fights this cancer cell successfully collects a sticker in his/her sticker album.

As mentioned in the introduction, entertainment should be the first aim of every game, so the first design premise over all others was to make the game really fun for the children. Regardless of how many paedagocial goals are achieved, all of these are of no real worth when the children won’t touch a boring game.

Something similar could be said about the art style. We oriented on modern animated movies, which usually choose an all age art style, making it suitable both for younger children and adolescents.

One very critical aspect, the look of the cancer cells, was also addressed in this way. The cells should not scare younger children, but, on the other hand, it would be wrong to show them as friendly pals. So, the following art style premise was set: ‘The enemies should look mean, but stupid. You have a chance against these dim-witted guys, so start fighting!’. The early and advanced drafts were shown to young cancer patients at the Universitätsklinikum des Saarlandes which pointed out clearly that these cancer cells don’t scare the children. More founded feedback is expected with the pilots of the ‘iManageCancer’ project.

## The serious game for kids step by step

**Figure 6.**
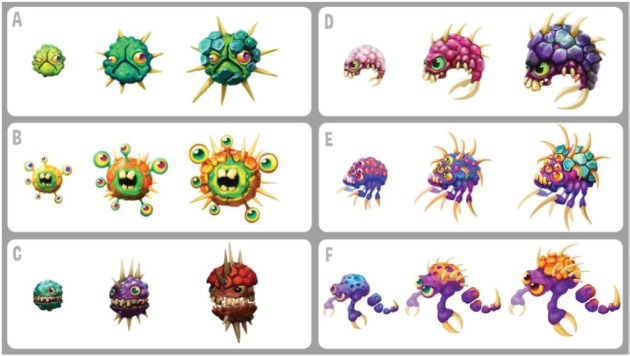
Picture shows several versions of the cancer cell Overview (2-D versions) from the serious game for children and adolescents.

Like the serious game for adults, the serious game for kids was designed in an iterative process (Figure 6). Many basic ideas were set in the beginning, but during development, we learned a lot about the very special use case of our game, and how that affects the game design.

### Choosing a game mode

When the serious game for kids is started, the player has to make a major decision first.

**Figure 7.**
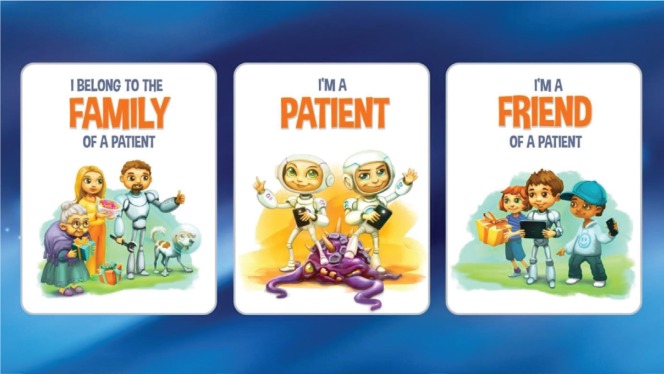
Screenshot shows the initial decision dialog for the game mode in the serious game for kids.

The screen shown in Figure 7 is designed to make it clear that the explorer/patient mode is reserved for children with cancer. In fact, misuse can’t be prohibited. The intention was also to make clear that everyone could be a supporter.

The selection screen is designed to show a very positive attitude and give the player confidence for the battle. This is why the patients are shown as potential winners here. The whole screen should work as an invitation to start the game.

### Selecting a level

**Figure 8.**
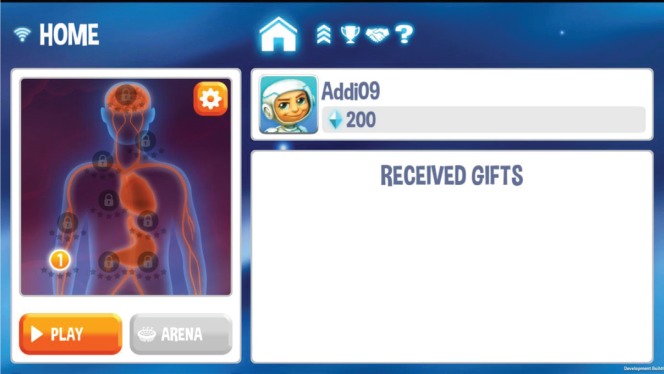
Screenshot shows the main level selection screen from the serious game for kids.

An image showing the silhouette of a human body with several inner organs is used as the level selection map (Figure 8). The figure has a unisex design, because players can be male or female.

Only the organs of the according game/level locations are visualised. The levels take place at five points of the human body: Capillary Vessels, Stomach, Sweat Glands, Heart and Brain.

From here, players choose the level they want to play or switch through the several menus which are listed in the top line. A high score list, some ship improvement menus and a help screen can be found there.

### Fighting the cancer cells

**Figure 9.**
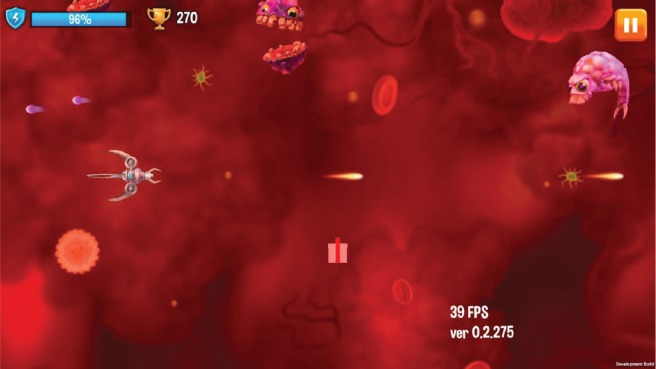
Screenshot shows a typical in-game situation from the serious game for children (supporter mode).

After selecting game mode and a level, the game starts. Figure 9 shows a typical game situation for a supporter.

The vessel travels through the capillary of the human body and meets different cancer cells. The interaction with the cancer cells is quite simple and reduced to shooting them.

In the middle of the screen is a gift box. They only appear in supporter mode and can be collected for the patients.

The game controls are designed to be as intuitive as possible. The player controls the vessel by simply touching the screen and moving the finger. The vessel shoots permanently and automatically. That makes the game simple for younger children, but still offering enough challenge because of the rising degrees of difficulty.

### The supporter mode

The supporter mode is made for family and friends, but technically everyone can become a ‘supporter’. A ‘supporter’ always has to choose a person to support. Travelling through the levels and fighting cancer cells makes the ‘supporters’ find special presents to transfer to their ‘patients’. The ‘supporter’ mode is split into a family and friends’ submode, which addresses the problem that family members may need a more extensive introduction to the game than friends.

When the patient players open one of the gift boxes distributed by a supporter player, they find shields and very effective extra weapons, which add a lot of fire power to their play.

## How the serious game for kids is integrated into ‘iManageCancer’

The serious game for kids is fully integrated into the ‘iManageCancer’ platform. Accounts for the platform work as accounts for the game—and vice versa.

This makes it mandatory that the serious games for kids have a login (which happens in the background once the device is logged in, so usually young kids do not see this) and all game results are sent to a server that realises all the social features.

There are mechanisms in place that allow controlling the access to the game via the platform, such as disabling supporters or reducing playtime. Parental control mechanisms are implemented to reduce parental concerns about the use of serious games.

The serious game for kids also reports game results to the iManageCancer PHR platform iphr.care. Here, activity and success in the game can be seen and are available for research. Also, an audit trail about the game process is stored on the platform. As all social features work via iphr.care, the data transfer is mandatory (The exception is that without connection to the Internet, the game can still be played and data exchange is stored meanwhile until the connection is back again.)

## Evolving design process in the game development phase

One main idea of the game was the social elements, but one result of reconsidering the final use case was that the players probably don’t have permanent access to the internet while playing the game. So, one of the first lessons learned about the game was that this needs to be addressed.

An offline mode was integrated. Once an account is initially logged in successfully, the game remembers this and will not ask for login again. So, a child usually doesn’t have to handle the login process. When there’s no connection to the Internet, the players get a message at the start of the game that they are offline and game results are stacked. The game itself remains fully playable, patient and supporter mode, shooter and match three.

A typical use case might be that the child in hospital plays with a device without an Internet connection, and when the parents come for a visit, they can establish a hotspot for a while to allow data exchange. After this transfer, game results and all the presents of grandparents and friends become available on the mobile devices of the kids.

Another learning outcome is that social aspects might turn into something negative when social support is not given by other people. When a game becomes easier through the help of others, then it is obviously more difficult (and probably very frustrating) when no support is given.

The game itself cannot solve the underlying problem, but the game can make sure that players do not feel weak in the game, because they have no supporters. Without any supporters, players find presents on their own. Also, mechanisms were implemented to distribute presents from the doctors to all players on the iphr.care platform. But the most important aspect of all might be that every player, with or without supporters, can contribute to the common goal to open the arena.

In the early stage of the design, there was a strong emphasis on the relation between the family and the young patients. This did not dignify enough the typical use case. In hospitals, the children will play for the fellow sufferer, the patients in the neighbouring bed, and this support is also highly welcome—which resulted in several user interface changes that make it easier to switch between patients.

Another aspect that was not considered sufficiently in the beginning was the differences in the skills of the players. The target group is not only young children and adolescents but there are also players who are handicapped by their disease. As a result, three different degrees of difficulty were added to the game. The more advanced the players are, the faster the cancer cell attacks.

The original idea for a serious game for kids was to introduce to the game some kind of paper hand-out. The feedback from the testing organisation, Tenovus, showed that test players do not understand the game mechanics and the meaning behind the game (here relatives like grandparents seem to have more comprehension problems than friends or siblings. Younger persons, all digital natives, may have a much easier and more intuitive approach to video games). So, a short tutorial with very short instructions was added that explains the game step by step. In a second iteration, we added a little tutorial character and some more extensive texts that add a little bit of narration.

As in the serious game for adults, the next step in the development will be to prove if all the enhancements lead to a measurable improvement regarding the mentioned issues.

## Conclusion

The serious game for adults offers colourful and engaging game play mechanics which keeps lifestyle and positive action at its core throughout. The design decisions made from the beginning have considered the need for positive feedback and opportunities to learn through exploration and vicarious experiences. To this end, the game has avoided penalising users for ineffective play, instead it aims to incentivise the development of critical problem-solving skills through mastery of the game mechanics and in-game item rewards.

The primary concept of the design is to reinforce the idea that engaging in healthy lifestyle management can help an individual overcome the challenges in their life caused by cancer and achieve a sense of wellbeing, a principle reinforced by the explicit mechanics of clearing challenges from their wellbeing meter in game.

The game for children and adolescents was designed to bring a positive attitude into the fight against cancer, make the patient’s role active, help them to focus on ‘the real enemy’ and provide some powerful social mechanics. The game went through several design changes that in particular improved the understandability of the game by adding a tutorial mode and improved user interface elements.

In the Tenovus testing sessions, both games showed deficits in the case of learning transfers as the major issue, and in both cases, this was addressed as described above. The games finally became ready for the pilot phase in the ‘iManageCancer’ project, with more than 100 patients involved. An evaluation workshop will be held after the pilot. The conclusions from this will not only lead to new insights into the efficacy of the serious games, they will also be used for another enhancement cycle.
